# Difficult Diagnosis of Spontaneous Intracranial Hypotension with Nausea and Lower Abdominal Pain as Main Complaints: A Case Report

**DOI:** 10.3390/reports7040115

**Published:** 2024-12-16

**Authors:** Misaki Yokoi, Tsuneaki Kenzaka, Mari Asano, Ryu Sugimoto, Hogara Nishisaki

**Affiliations:** 1 Department of Internal Medicine, Hyogo Prefectural Tamba Medical Center, Tamba 669-3495, Hyogo, Japan; misaki.yokoi1108@gmail.com (M.Y.); izkmr627@outlook.jp (M.A.); ryuy153145@yahoo.co.jp (R.S.); honssk-d@sanynet.ne.jp (H.N.); 2 Division of Community Medicine and Career Development, Kobe University Graduate School of Medicine, Kobe 652-0032, Hyogo, Japan

**Keywords:** spontaneous intracranial hypotension, lower abdominal pain, dysmenorrhea, cerebrospinal fluid depletion, intrabladder radioisotope accumulation, case report

## Abstract

**Background and Clinical Significance**: Symptoms of spontaneous intracranial hypotension include orthostatic headaches due to decreased cerebrospinal fluid (CSF) levels. Here, we present a 24-year-old female admitted to an obstetrics and gynecology department with primary complaints of lower abdominal pain and dysmenorrhea with subsequent diagnosis of spontaneous intracranial hypotension (SIH). **Case Presentation**: The patient had experienced nausea and lower abdominal pain independent of her menstrual cycle 5 days before admission, for which she visited the emergency department 3 days later. On admission, her symptoms were temporarily relieved by administering analgesics; thus, she was discharged. However, later, the symptoms worsened. Consequently, she returned to the emergency department for further evaluation, including blood tests, imaging, and endoscopy, which revealed no nausea- or abdominal pain-related organic abnormalities. On day 10, she developed a headache, aggravated by lying in the supine position and improved by sitting. Additional history revealed a diagnosis of SIH owing to the worsening abdominal pain in the supine position. An ^111^In CSF cavity scintigram showed no spinal fluid leakage; early intrabladder radioisotope (RI) accumulation was observed, and the residual 24 h CSF cavity RI was >30%. At a referral specialist hospital, an epidural saline infusion test was performed, which improved her headache and lower abdominal pain. Blood patch therapy improved her lower abdominal pain, headache, and dysmenorrhea. **Conclusions**: The final diagnosis was SIH, with symptoms attributed to CSF depletion. The patient also experienced rare paradoxical postural-related headaches and lower abdominal pain, aggravated by lying in the supine position, contributing to the final diagnosis.

## 1. Introduction and Clinical Significance

Spontaneous intracranial hypotension (SIH) is a condition in which the cerebrospinal fluid (CSF) level is reduced through mechanisms such as decreased production, increased absorption and leakage, or both [1]. This condition manifests through various primary symptoms, including headache, neck pain, dizziness, tinnitus, visual dysfunction, malaise, and fatigue [1]. The frequency of this condition is approximately 5 per 100,000 people, and it is more common in female individuals than in male individuals [2]. Owing to the nature of the various symptoms, this condition is often misdiagnosed as chronic headache, cervical spondylosis, or depression. Furthermore, although the International Classification of Headache of the International Headache Society specifies diagnostic criteria for idiopathic low-CSF headache [3], the disease remains underrecognized by many medical professionals.

SIH is also associated with a range of other symptoms, including cerebral neurological symptoms, impaired consciousness, anhedonia, cerebellar ataxia, Parkinson’s syndrome, and gait disturbance [4]. Other related symptoms include dementia, memory impairment, pain and numbness in the upper limbs, cystorectal disturbances, milk secretion, nausea and vomiting, interscapular pain, and back pain [4].

However, there have been no prior reports of abdominal pain as a primary symptom. Headache and other symptoms are characteristically aggravated when sitting or standing [3]. However, in this report, we present a rare case of SIH where lower abdominal pain and dysmenorrhea were the main symptoms, which improved while sitting or standing and worsened in the supine position.

## 2. Case Presentation

A 24-year-old Japanese female individual arrived at our hospital with nausea and lower abdominal pain, which had developed five days before admission, independent of her menstrual cycle. Two days before admission, the patient was brought to the emergency department because of the lower abdominal pain. The cause of this abdominal pain remained unidentified, although her symptoms were temporarily relieved with the administration of analgesics, and she was discharged. However, her symptoms subsequently worsened, prompting her to return to the emergency department, where she was admitted for further investigation.

The patient had no history of abdominal surgery but had been receiving treatment at an obstetrics and gynecology clinic for the past six months for dysmenorrhea. She had been taking low-dose pills (levonorgestrel/ethinylestradiol), which were discontinued eight days before admission because of elevated liver enzyme levels. Additionally, she used ad hoc antiemetics and painkillers (diclofenac tablets) as needed. Her social history was unremarkable.

Upon arrival, the patient was alert and fully conscious. Her vital signs were as follows: temperature, 37.1 °C; blood pressure, 122/59 mmHg; pulse rate, 63 beats/min; respiratory rate, 18 breaths/min; and oxygen saturation, 99% (room air). Physical examination revealed tenderness over the entire lower abdomen but no peritoneal irritation symptoms or skin rash, whereas neurological examinations revealed no abnormalities. Blood tests revealed elevated levels of liver enzymes—specifically, aspartate aminotransferase at 74 U/L and alanine aminotransferase at 121 U/L—but no other specific findings. Contrast-enhanced computed tomography of the abdomen did not reveal any organic disease that could explain the abdominal pain, including in the abdominal cavity, skin, soft tissue, or bone. Immunological tests, such as those for the detection of antineutrophil cytoplasmic and antinuclear antibodies, and endocrine tests, including those for the detection of adrenocorticotropic hormone, cortisol, and thyroid function, showed no abnormal findings. The liver dysfunction improved spontaneously. Other examinations, including obstetric and gynecological assessments, transvaginal and abdominal ultrasonography, simple and contrast-enhanced magnetic resonance imaging (MRI) of the pelvic region, upper gastrointestinal endoscopy, and colonoscopy, revealed no organic abnormalities that could explain the nausea or abdominal pain.

On day 10 of admission, the patient developed a headache that was aggravated in the supine position and improved in the sitting position. A more detailed interview with the patient revealed that her lower abdominal pain was aggravated in the supine position. Suspecting abnormal CSF, a lumbar puncture was performed. The CSF was colorless and clear at an initial pressure of 8 cm H_2_O. No increase in protein levels or decrease in sugar levels was observed in the CSF, and bacterial cultures were negative. Given the positional nature of the symptoms, such as exacerbation when lying down and resolution while sitting, SIH was suspected, prompting additional investigations.

**Figure 1:**
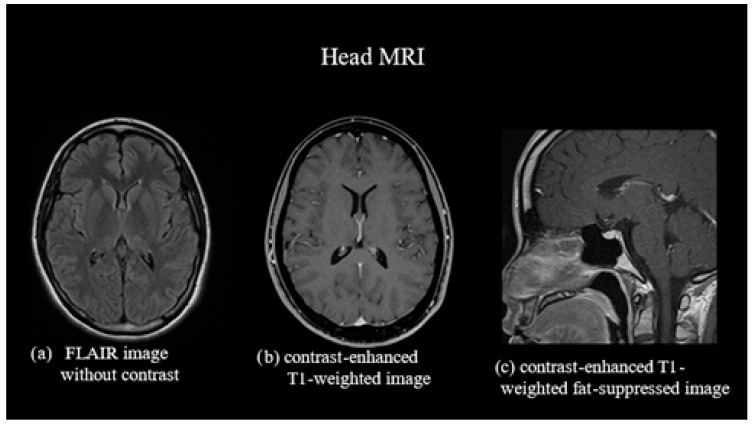
Magnetic resonance imaging (MRI) of the head. (**a**) Non-contrast fluid-attenuated inversion recovery image showing no subdural fluid accumulation. (**b**) Contrast-enhanced T1-weighted image showing no diffuse dural thickening. (**c**) Contrast-enhanced T1-weighted fat-suppressed image showing no dilation of the epidural venous plexus. FLAIR, fluid-attenuated inversion recovery.

**Figure 2:**
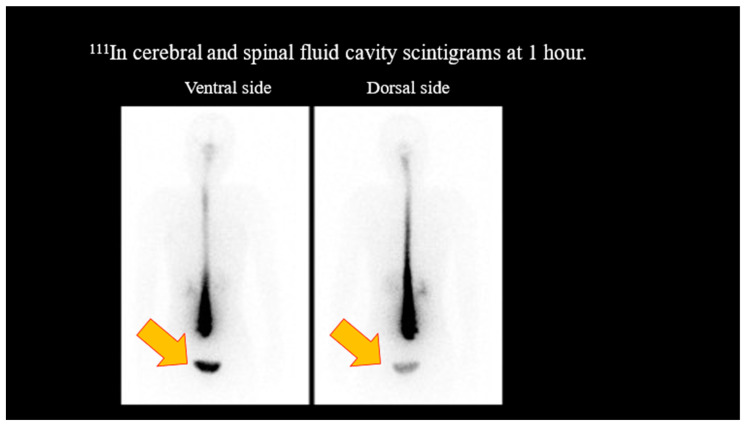
^111^In cerebrospinal fluid cavity scintigrams at 1 h. No spinal fluid leakage was observed. However, early accumulation of radioisotopes in the bladder was observed, suggesting spinal fluid leakage.

**Figure 3:**
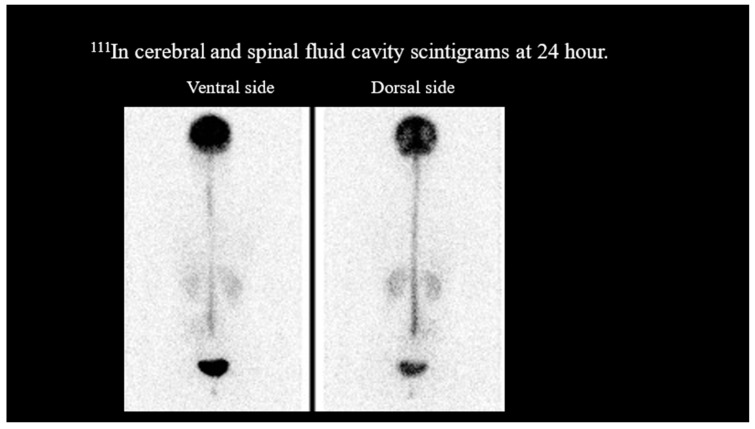
^111^In cerebrospinal fluid cavity scintigrams at 24 h. The residual radioisotope in the cerebrospinal fluid space was 15% at 24 h (normal, >30%), suggesting spinal fluid leakage.

Non-contrast- and contrast-enhanced MRI of the head showed no dural edema. Contrast-enhanced T1-weighted images revealed no diffuse dural thickening or dilation of the epidural venous plexus (Figure 1). However, ^111^In cerebroventricular and spinal fluid space scintigraphy showed early cystic accumulation at 1 h (Figure 2), although spinal fluid leakage was not evident. Accumulation was also observed after approximately 6 h, and the residual CSF cavity radioisotope (RI) after 24 h was 15% (Figure 3), lower than the normal value of >30%.

SIH was strongly suspected, leading to the patient’s referral to a specialist. The headache and lower abdominal pain improved after an epidural saline infusion test was performed at the specialist hospital. Symptoms, such as headache and lower abdominal pain, were resolved after blood patch therapy, and the patient’s dysmenorrhea also improved. Based on these findings, the patient was diagnosed with SIH, and her lower abdominal pain, headache, and dysmenorrhea were recognized as symptoms associated with this diagnosis. During the subsequent three-year observation period, she did not have any recurrence of symptoms.

## 3. Discussion

Here, we present a case of SIH in which lower abdominal pain and dysmenorrhea were the main symptoms. To the best of our knowledge, this is the first report highlighting these symptoms as the main manifestations of SIH. In this case, headache and lower abdominal pain were exacerbated in the supine position, which is atypical.

**Figure 4:**
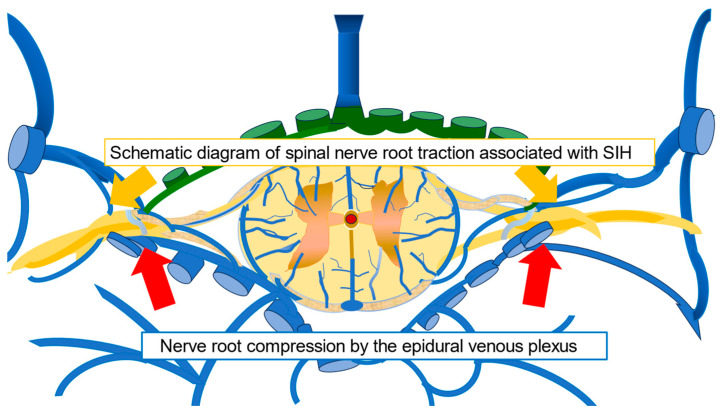
Schematic diagram of spinal nerve root traction associated with SIH and nerve root compression by a dilated epidural venous plexus. Original drawing based on ref. [6].

The underlying pathology of SIH is presumed to include cranial nerve traction due to a downward shift in the brain and a compensatory increase in intracranial blood volume due to CSF depletion [1]. Additionally, endocrine disorders may develop in the chronic phase because of abnormal pituitary function [5], and dysmenorrhea is considered as a symptom associated with SIH. Furthermore, traction of the cervical nerve roots and compression of the nerve roots by the dilated epidural venous plexus have been previously reported as mechanisms underlying the development of upper limb symptoms [6]. In the present case, abdominal pain was observed in an area consistent with nerve movement. Although MRI results revealed no clear abnormality, we inferred that the lower abdominal pain was due to compression of the dorsal root of the thoracic spinal cord by the dilated epidural venous plexus. Figure 4 shows a schematic diagram hypothesizing the relationship between spinal nerve root traction associated with SIH and nerve root compression by a dilated epidural venous plexus.

Another unusual finding in this case of SIH is the exacerbation of associated headache and lower abdominal pain in the supine position. Orthostatic headache, which appears and worsens in the sitting position, is typically associated with SIH [3]. However, some patients report experiencing headaches when rising or in the supine position [7]. Notably, 23% of patients have been documented to not experience typical orthostatic headache [2].

Incidences of headache exacerbation in the supine position, as in the present case, have been reported as “paradoxical postural headaches” [7]. The mechanism believed to underlie this occurrence involves the dilatation of the cerebral venous sinuses and intracranial veins, which is aggravated in the supine position [7]. Subdural fluid accumulation, diffuse dural thickening, and dilation of the epidural venous plexus are characteristic findings of SIH on head MRI and are observed in 83, 61, and 75% of patients, respectively [8]. Although MRI results did not reveal any clear abnormalities, we speculated that the worsening headache in the supine position in this case was due to a compensatory increase in intracranial blood volume.

The first-line treatment for SIH is the lumbar epidural blood patch. It is believed that the blood patch works by initially tamponading the dural leak, followed by fibrin deposition and scar formation [9]. The success rate of the blood patch has been reported to be 64% [10].

## 4. Conclusions

We present the case of a patient with SIH in which lower abdominal pain and dysmenorrhea were the main symptoms. Notably, the patient exhibited rare paradoxical postural symptoms of headache and lower abdominal pain, which were aggravated in the supine position. The patient’s history, highlighting how changes in position aggravated her symptoms, played a crucial role in the diagnosis of SIH.

## Data Availability

All data generated or analyzed during this study are included in the published article. Further inquiries can be directed to the corresponding author.
